# Comparison of replicating and nonreplicating vaccines against SARS-CoV-2

**DOI:** 10.1126/sciadv.abm8563

**Published:** 2022-08-24

**Authors:** Haley E. Mudrick, Shane Massey, Erin B. McGlinch, Brian J. Parrett, Jack R. Hemsath, Mary E. Barry, Jeffrey D. Rubin, Chisom Uzendu, Michael J. Hansen, Courtney L. Erskine, Virginia P. Van Keulen, Aleksandra Drelich, Joseph A. Panos, Madiha Fida, Gina A. Suh, Tobias Peikert, Matthew S. Block, Chien-Te Kent Tseng, Gloria R. Olivier, Michael A. Barry

**Affiliations:** ^1^ Molecular Pharmacology and Experimental Therapeutics (MPET) Graduate Program, Mayo Clinic, Rochester, MN, USA.; ^2^ Center of Biodefense and Emerging Disease, University of Texas Medical Branch, Galveston, TX, USA.; ^3^ Graduate Research Education Program (GREP), Mayo Clinic, Rochester, MN, USA.; ^4^ Virology and Gene Therapy (VGT) Graduate Program, Mayo Clinic, Rochester, MN, USA.; ^5^ Department of Medicine, Division of Infectious Diseases, Mayo Clinic, Rochester, MN, USA.; ^6^ Department of Immunology, Mayo Clinic, Rochester, MN, USA.; ^7^ Department of Microbiology and Immunology, University of Texas Medical Branch, Galveston, TX, USA.; ^8^ Rehabilitation Medicine Research Center, Musculoskeletal Gene Therapy Research Laboratory, Mayo Clinic Medical Scientist Training Program, Mayo Clinic, Rochester, MN, USA.; ^9^ Department of Medical Oncology, Mayo Clinic, Rochester, MN, USA.; ^10^ Department of Medicine, Division of Pulmonary Care, Mayo Clinic, Rochester, MN, USA.; ^11^ Institutional Office of Regulated Nonclinical Studies, University of Texas Medical Branch, Galveston, TX, USA.; ^12^ Mayo Clinic Ventures, Mayo Clinic, Rochester, MN, USA.; ^13^ Department of Molecular Medicine, Mayo Clinic, Rochester, MN, USA.

## Abstract

Most gene-based severe acute respiratory syndrome coronavirus 2 (SARS-CoV-2) vaccines are nonreplicating vectors. They deliver the gene or messenger RNA to the cell to express the spike protein but do not replicate to amplify antigen production. This study tested the utility of replication in a vaccine by comparing replication-defective adenovirus (RD-Ad) and replicating single-cycle adenovirus (SC-Ad) vaccines that express the SARS-CoV-2 spike protein. SC-Ad produced 100 times more spike protein than RD-Ad and generated significantly higher antibodies against the spike protein than RD-Ad after single immunization of Ad-permissive hamsters. SC-Ad–generated antibodies climbed over 14 weeks after single immunization and persisted for more than 10 months. When the hamsters were challenged 10.5 months after single immunization, a single intranasal or intramuscular immunization with SC-Ad-Spike reduced SARS-CoV-2 viral loads and damage in the lungs and preserved body weight better than vaccination with RD-Ad-Spike. This demonstrates the utility of harnessing replication in vaccines to amplify protection against infectious diseases.

## Teaser

Replicating single-cycle adenovirus SARS-CoV-2 vaccine is more potent than matched replication-defective vaccine.

## INTRODUCTION

Nearly every vaccine technology has been deployed to combat severe acute respiratory syndrome coronavirus 2 (SARS-CoV-2) [reviewed in (*1*)]. mRNA vaccines advanced most quickly in the United States with approval from the Food and Drug Administration. While these advanced vaccines show promise in the fight against coronavirus disease 2019 (COVID-19), they overlook two opportunities to improve protection.

First, most COVID-19 vaccines are administered into the muscle at a site where SARS-CoV-2 never enters the body. Second, most COVID-19 vaccines are replication-defective (RD) and miss out on the opportunity to amplify antigen transgenes, amplify antigen production, and amplify immune responses against SARS-CoV-2.

We developed single-cycle adenovirus (SC-Ad) vectors, which replicate their antigen genes 10,000 times in human cells, but that do not produce infectious progeny virions (*2*–*5*). This contrasts with RD-Ad vectors (such as the Johnson & Johnson human Ad26 vaccine, the ChAdOx1 vaccine from Oxford/AstraZeneca, and the Russian Sputnik V Ad26 and Ad5 vaccines) that do not replicate antigen genes or produce infectious progeny virions.

We generated RD-Ad and SC-Ad vectors expressing the wild-type original SARS-CoV-2 spike protein and compared their abilities to produce the spike protein and generate immune responses in rodents. We used these vaccines to compare the utility of mucosal intranasal (IN) immunization and systemic intramuscular (IM) immunization to generate immune responses in systemic and mucosal compartments. Last, we compared the ability of a single IN or a single IM vaccination with these vaccines to protect against respiratory infection by SARS-CoV-2.

## RESULTS

### SC-Ad and RD-Ad SARS-CoV-2 vaccines

**Fig. 1.**
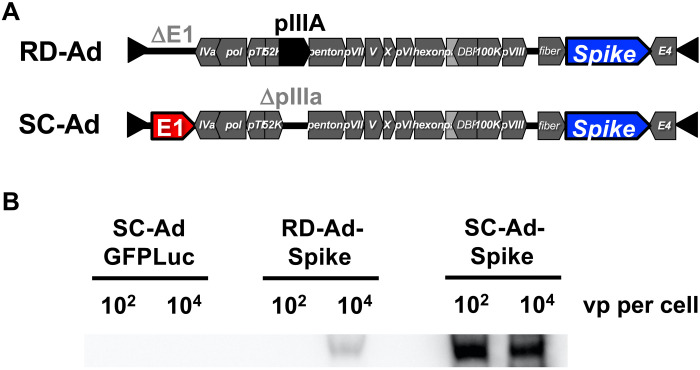
Replication-defective and single-cycle adenoviruses expressing SARS-CoV-2 Spike. (**A**) Schematics of the RD-Ad-Spike vaccine construct as compared to the SC-Ad-Spike vaccine construct. In the RD-Ad-Spike vector, the E1 protein has been removed and the SARS-CoV-2 spike protein has been inserted. In the SC-Ad-Spike vector, the pIIIA protein has been removed instead of the E1 protein, and the SARS-CoV-2 spike protein has been inserted. (**B**) Western blot of human A549 lung cells infected with SC-Ad vector with GFP-Luciferase (SC-Ad GFPLuc), RD-Ad-Spike, or SC-Ad-Spike at doses of 10^2^ and 10^4^ viral particles per cell. All cells were harvested 24 hours after infection, and equal fractions of the cells were subjected to SDS-PAGE and a Western blot was performed against the SARS-CoV-2 spike protein.

A codon-optimized complementary DNA (cDNA) encoding the original wild-type spike protein from the 2019-nCoV HKU-SZ-002a 2020 isolate was inserted into RD-Ad and SC-Ad adenovirus serotype 6 (Ad6) vectors. The cDNA encodes the original 2019 SARS-CoV-2 native spike protein with its natural secretory leader and does not include proline mutations to alter the spike structure (*6*–*8*). This cDNA was inserted into a cytomegalovirus (CMV) expression cassette and was used to generate human Ad6 vectors RD-Ad6-spike and SC-Ad6-spike, respectively (Fig. 1A).

These vectors were tested for spike protein expression by infection of human A549 lung cells at varied multiplicities of infection (MOIs) (Fig. 1B). Western blot on cells harvested 24 hours after infection demonstrated that both vectors produced spike protein; however, RD-Ad-Spike only generated detectable protein with 10^4^ virus particles (vp) per cell, but not with 100 vp per cell. In contrast, SC-Ad-Spike produced protein with 100 or more vp per cell with higher expression than RD-Ad at each dose.

### Levels and duration of spike antibody production after single immunization

Human Ad6 replicates its genome up to 100,000-fold in human cells (*2*, *9*). SC-Ad replicates DNA identically to replication-competent (RC)–Ad (*2*). Mice, however, do not support replication of human Ads (*10*) and cannot manifest the transgene amplification via DNA replication that might be observed in Ad-permissive humans. In contrast, Syrian hamsters are permissive for infection by Ad6 (*11*, *12*). While they are permissive, Syrian hamster cells allow only 350-fold replication of Ad6 DNA after infection in contrast to higher levels in human cells (*13*). Thus, hamsters can manifest the effects of SC-Ad transgene replication but will likely underrepresent the replication effect that may occur in humans by a factor of 10 or more.

**Fig. 2.**
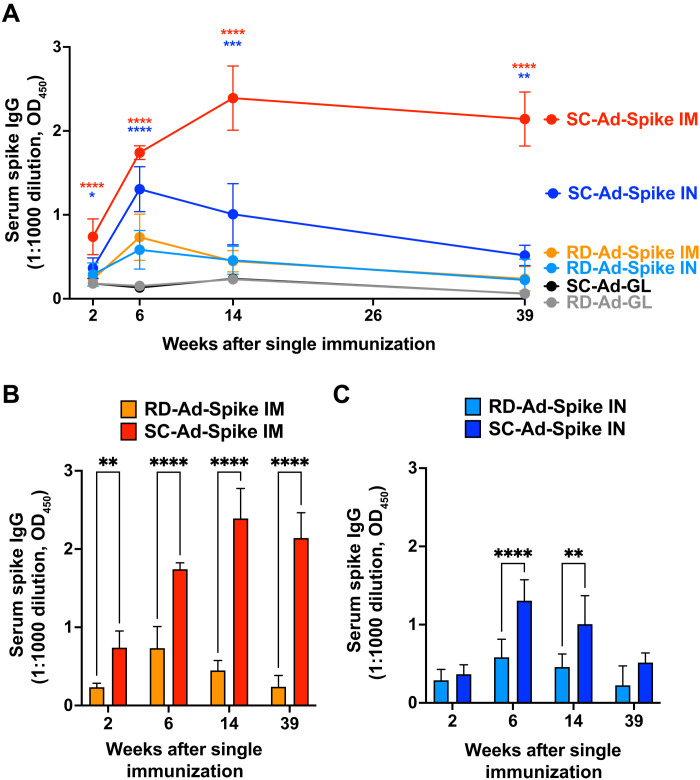
Kinetics of spike antibody production by RD-Ad and SC-Ad vaccines after a single IN or IM vaccination. Groups of five male Syrian hamsters were immunized at a dose of 10^9^ vp, and serum was collected at weeks 2, 6, 14, and 39 after single immunization. (**A**) Serum was used at 1:1000 dilution to test for SARS-CoV-2 spike IgG antibodies by enzyme-linked immunosorbent assay (ELISA). (**B** and **C**) Comparison of serum spike IgG antibodies in Syrian hamsters immunized with RD-Ad-Spike and SC-Ad-Spike by IM (B) and IN (C) routes of administration, analyzed by ELISA at 1:1000 serum dilution. Error bars represent SDs [*P < 0.05, **P < 0.01, ***P < 0.001, and ****P < 0.0001, by one-way analysis of variance (ANOVA)].

Male and female Syrian hamsters were immunized once with 10^9^ vp of RD-Ad-Spike and SC-Ad-Spike by IN and IM routes or with negative control RD- and SC-Ad vectors expressing green fluorescent protein (GFP)–Luciferase (GL). After a single immunization, SC-Ad-Spike generated significantly higher spike antibody levels than RD-Ad-GL or SC-Ad-GL or when compared to RD-Ad-Spike (Fig. 2 and fig. S1).

Antibody production in sera peaked at week 6 for the RD-Ad vectors and SC-Ad-Spike by the IN route, whereas IM SC-Ad-Spike reached a plateau of spike-binding antibodies at week 14 that largely persisted through the end of the study (Fig. 2A). SC-Ad-Spike generated higher immunoglobulin G (IgG) than RD-Ad-Spike at all time points through 39 weeks after single immunization (Fig. 2B). SC-Ad-Spike by the IN route generated significantly higher IgG in sera than RD-Ad-Spike at 6 weeks and 14 weeks, but not at weeks 2 and 39 after single immunization (Fig. 2C).

Week 6 sera were assayed for SARS-CoV-2 neutralizing antibodies with the cPass Neutralization Antibody Detection kit. This assay measures the ability of antibodies to bind to the receptor binding domain (RBD) of spike and block its ability to bind to angiotensin-converting enzyme 2 (ACE2) on enzyme-linked immunosorbent assay (ELISA) plates. By this assay, SC-Ad-Spike generated significant inhibition by the IN and IM routes at this early time point after single immunization (fig. S2). RD-Ad–vaccinated hamsters failed to generate significant spike RBD inhibition at any dilution of sera at 6 weeks.

Many mutations have arisen in SARS-CoV-2 as different variants have been selected in the human population. For example, the spike protein from SARS-CoV-2 alpha variant B.1.1.7 contains H69del, V70del, Y144del, N501Y, A570D, D614G, and P681H mutations. The beta B.1.351 variant has K417N, E484K, N501Y, and D614G mutations. The gamma P.1 variant spike protein bears K417T, E484K, and N501Y mutations (*14*–*16*). The spike proteins from the newer delta and omicron variants have an even more diverse array of mutations in spike.

**Fig. 3.**
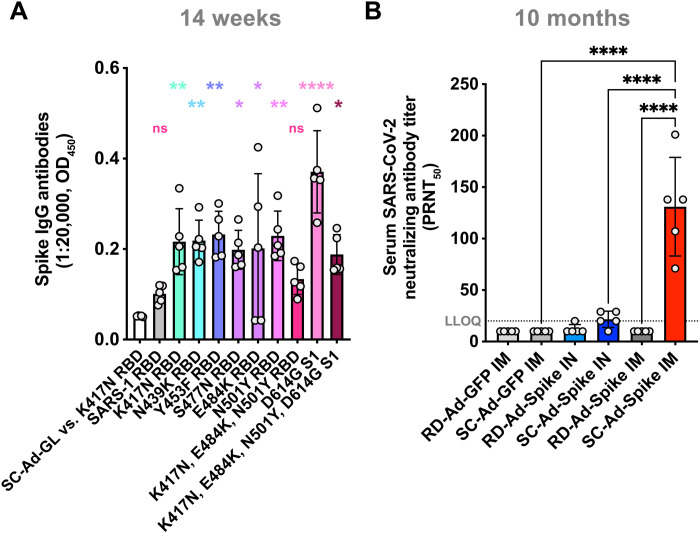
Functionalities of antibodies produced by single RD-Ad-Spike and SC-Ad-Spike immunizations. (**A**) Week 14 sera from SC-Ad-Spike or SC-Ad-GL IM hamsters were analyzed for binding to variant RBDs and S1 proteins by ELISA at 1:1000, 1:10,000, and 1:20,000 dilutions. All proteins are RBDs except the D614G S1 and the K417N, E484K, N501Y, D614G S1 subunit proteins. Antibodies generated by SC-Ad-GL showed only background binding to all the variants. This is exemplified by the low OD_450_ for “SC-Ad-GL vs. K417N” in (A). (**B**) Sera were collected from the hamsters from Fig. 2 10 months after single immunization and were assayed for SARS-CoV-2 neutralizing antibodies. The limit of detection of the assay was defined as 1 plaque per well. The lower limit of quantification (LLOQ) was 20 for this assay. Samples below 20 are shown below the LLOQ. Error bars represent SDs (*P < 0.05, **P < 0.01, ***P < 0.001, and ****P < 0.0001). ns, not significant.

The SC-Ad vaccine expresses the original 2019 spike protein without any of these mutations and without the two proline mutations that modify how spike is displayed. To test whether SC-Ad-Spike generates any degree of cross-reactivity, week 14 sera from SC-Ad-GL– and SC-Ad-Spike–immunized hamsters were tested by ELISA against a subset of the mutations described above. Sera were tested against variant spike RBDs (amino acids 319 to 541) and against larger spike S1 proteins (amino acids 16 to 685). An RBD protein from the original 2007 SARS virus (referred to as “SARS-1”) was included as a divergent control target protein. ELISAs performed with 1:20,000 dilutions of week 14 sera demonstrated significant binding by samples from SC-Ad-Spike–immunized animals to all the variant RBDs and S1 proteins when compared to SC-Ad-GL samples (see SC-Ad-GL vs. K417N as an exemplar in Fig. 3A and fig. S3).

When the samples were tested at 1:1000 dilutions of sera, antibody binding to all RBD and S1 variants was significant (fig. S3). With 20-fold more dilute sera (1:20,000 dilutions), antibody binding remained significant against all spike RBD variants [*P* < 0.05 to 0.0001 by one-way analysis of variance (ANOVA)] except when tested against the triple mutant K417N, E484K, N501Y RBD (Fig. 3A). While antibody binding to this small RBD was lost, the sera still bound significantly to the larger S1 subunit protein with the same mutations (K417N, E484K, N501Y) that also included the D614G mutation. This was not unexpected, since the larger spike S1 protein has more than 400 additional amino acids and many more epitopes for polyclonal antibody binding.

Therefore, SC-Ad-Spike expressing the original, unmodified spike sequence from the 2019 isolate of SARS-CoV-2 was able to generate cross-reactivity against most point mutations in the RBD domain, but that cross-reactivity is lower with more complex combinations.

### Long-term protection against SARS-CoV-2 after single mucosal or systemic immunization

The male hamsters from Figs. 2 and 3 were kept for 10 months after a single immunization. At this time point, none of the sera from RD-Ad-GL, SC-Ad-GL, or RD-Ad-Spike IM–immunized animals had detectable SARS-CoV-2 (USA_WA1/2020) neutralizing antibody titers. All were below the lower limit of quantification (LLOQ) of 20 (Fig. 3B). Four of five SC-Ad-Spike IN–immunized animals had reciprocal titers of 20 or higher, while only one animal in the RD-Ad-Spike group had a neutralizing titer above 20. In contrast, all animals in the SC-Ad-Spike IM group had SARS neutralizing titers above 20. These levels were notable given that they occurred more than 10 months after a single immunization.

**Fig. 4.**
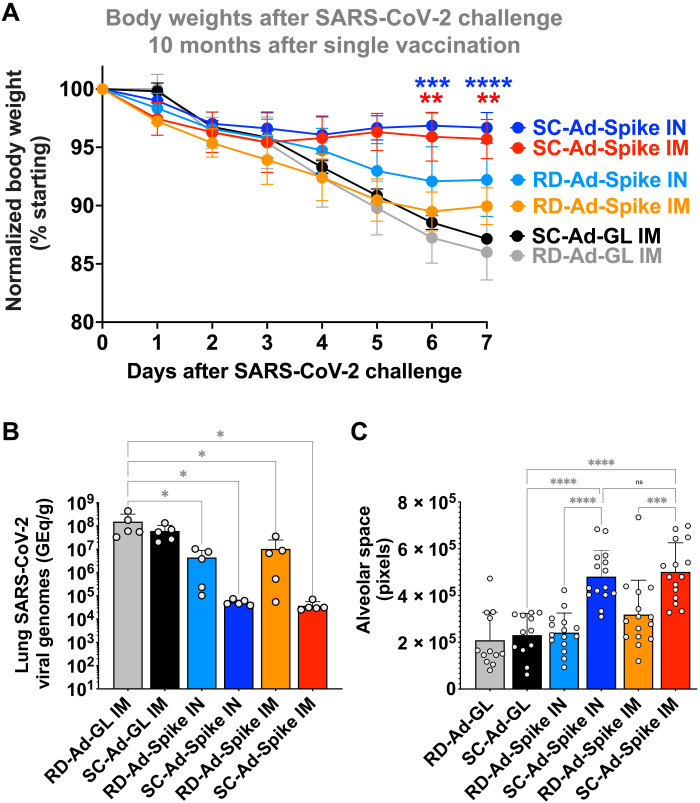
SARS-CoV-2 challenge of vaccinated animals 10 months after single immunization. The male Syrian hamsters from Fig. 2 were challenged intranasally with SARS-CoV-2 2020 WA 10 months after single vaccination. (**A**) Changes in body weight after challenge. Comparisons versus SC-Ad-GL are shown by two-way ANOVA. SC-Ad-Spike IM was also significantly different than RD-Ad-Spike IM on days 5 and 6 (*P* < 0.05 and *P* < 0.0001, respectively). SC-Ad-Spike IN was also significantly different than RD-Ad-Spike IN on day 6 (*P* < 0.01). RD-Ad-Spike by IN or IM routes was not significant when compared to RD-Ad-GL at any time point by this analysis. (**B**) SARS-CoV-2 genomic viral loads in lungs 7 days after challenge determined by quantitative reverse transcription polymerase chain reaction (qRT-PCR). (**C**) Lung alveolar space on day 7 after challenge calculated in MATLAB from images provided in Fig. 5. Three images per lung were quantified, yielding the indicated 15 data points per group (3 images × 5 animals per group). Comparisons are by one-way ANOVA. Error bars represent SDs (*P < 0.05, **P < 0.01, ***P < 0.001, and ****P < 0.0001).

The animals were challenged intranasally with 10^6^ median tissue culture infectious dose (TCID_50_) SARS-CoV-2 (USA_WA1/2020) and were monitored for 7 days. Control animals vaccinated with RD-Ad-GL and SC-Ad-GL viruses lost up to 15% of their body weight by day 6 (Fig. 4A). Animals immunized with RD-Ad-Spike by the IM or IN route lost less weight than controls. Weight loss was significantly less in the SC-Ad-Spike IM than in the RD-Ad-Spike IM group on days 5 and 6 (*P* < 0.05 and *P* < 0.0001 by two-way ANOVA, respectively). Weight loss in SC-Ad-Spike IN–immunized hamsters was also significantly less than in RD-Ad-Spike IN–immunized animals on day 6 (*P* < 0.01).

All vaccinated groups had significantly lower SARS-CoV-2 viral genome levels in their lungs than controls at 7 days after challenge (Fig. 4B). The RD-Ad vaccines suppressed SARS-CoV-2 viral loads from 10- to 1000-fold with high variation. In contrast, the viral loads in the lungs of the SC-Ad-Spike–immunized animals were all reduced below 30,000 genome equivalents (GEq)/g and were lower than the levels in control or in RD-Ad-Spike–vaccinated animals. While there were obvious differences in viral suppression by SC-Ad, the high variation in the RD-Ad-Spike groups made their comparison to SC-Ad vaccines not significantly different by ANOVA. SARS-CoV-2 viral loads in the lungs were not significantly different between SC-Ad-Spike IM and IN groups.

**Fig. 5.**
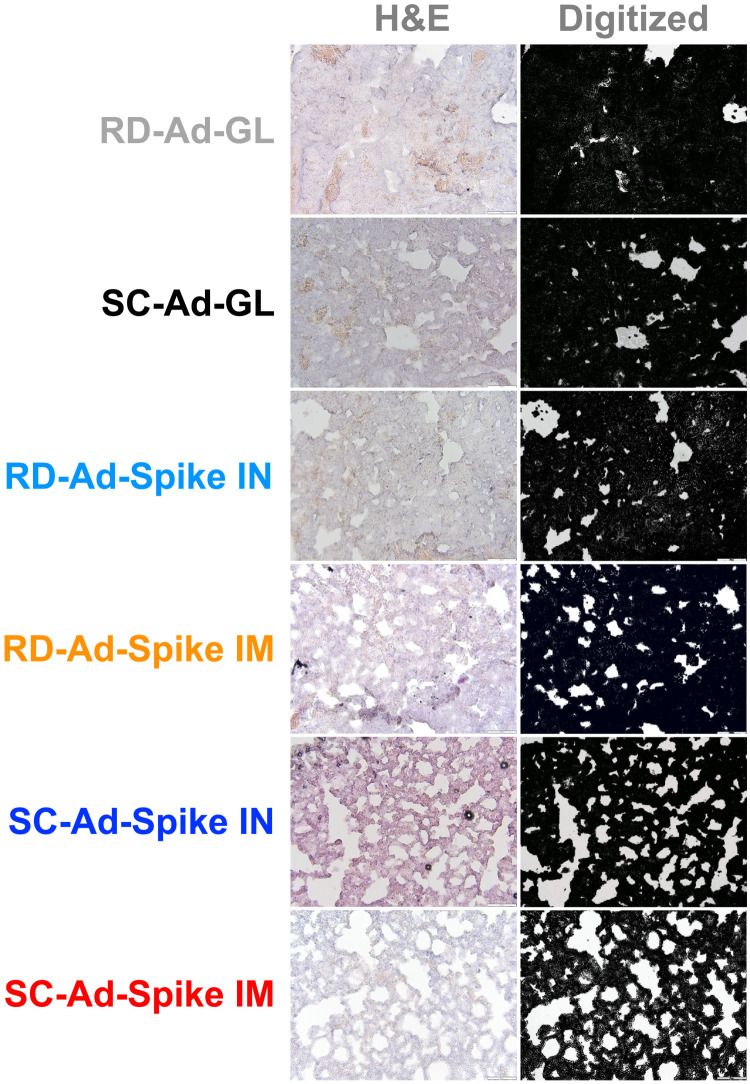
Lung sections from SARS-CoV-2–challenged animals. Lungs were collected 7 days after challenge. These were sectioned and stained with hematoxylin and eosin (H&E) (left panels). Representative images are shown. Three separate images from each animal were digitized (right panels), and the number of pixels in alveoli was quantified using MATLAB.

Lungs were collected 7 days after challenge and were also examined by hematoxylin and eosin (H&E) staining for pathology due to SARS-CoV-2 infection (Fig. 5). RD-Ad-GL and SC-Ad-GL control animals had substantial immune infiltrates and damage within lungs. Most RD-Ad-Spike animals had similar damage, but to slightly lower degrees. In contrast, the lungs in SC-Ad-Spike-immunized hamsters suffered less damage and maintained significantly more open alveoli in their lung sections (*P* < 0.0001 by one-way ANOVA) (Fig. 4C). SC-Ad-Spike IN was significantly better than RD-Ad-Spike IN (*P* < 0.0001) and SC-Ad-Spike IM was significantly better than RD-Ad-Spike IM (*P* < 0.001). Alveolar size in SC-Ad-Spike IM and IN animals was not significantly different.

**Fig. 6.**
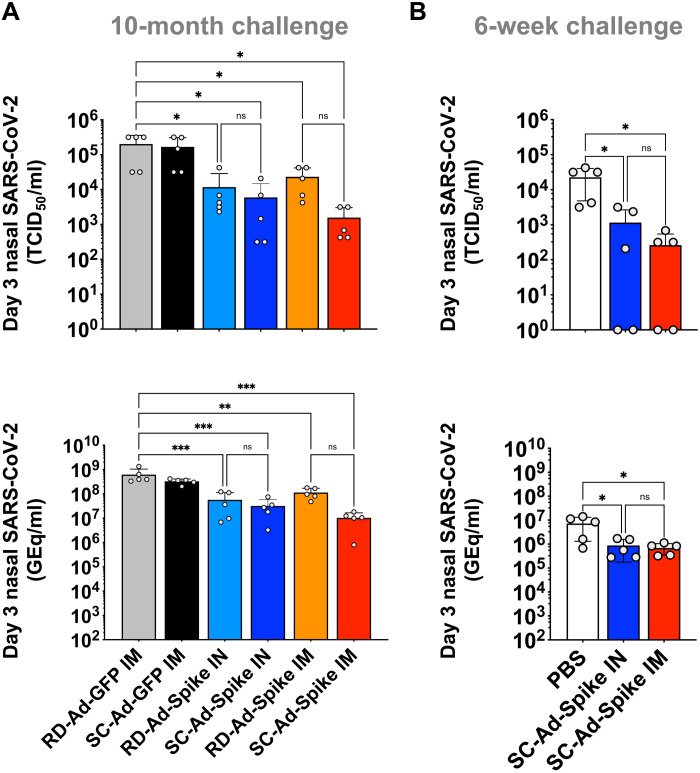
SARS-CoV-2 virus in nasal samples after challenge at 6 weeks or 10 months after single immunization. Nasal samples were collected from 6-week and 10-month challenge vaccine study animals, and SARS-CoV-2 was titered by qRT-PCR (genomic) and TCID_50_ assay. Error bars represent SDs (*P < 0.05, **P < 0.01, ***P < 0.001, and ****P < 0.0001, by one-way ANOVA).

The RD-Ad-Spike and SC-Ad-Spike vaccines all reduced SARS-CoV-2 viral loads in nasal samples when the animals were challenged 10 months after vaccination as assessed by quantitative reverse transcription polymerase chain reaction (qRT-PCR) or by TCID_50_ assays (Fig. 6 and fig. S4). SC-Ad viral loads were lower than the levels in RD-Ad–vaccinated animals but did not reach significance. These results showed favorable reductions in nasal SARS-CoV-2 viral loads by all the vaccines, but not sterilizing immunity. The lack of effects on nasal viral loads were markedly different than the reduced viral loads observe in lungs between RD-Ad and SC-Ad groups.

### Short-term protection against SARS-CoV-2 after single mucosal or systemic immunization

**Fig. 7.**
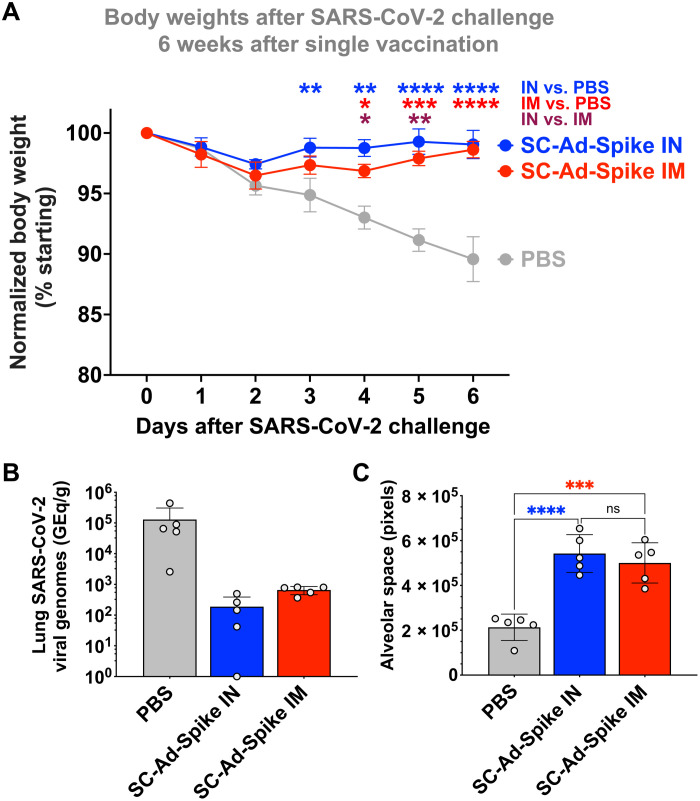
SARS-CoV-2 challenge of vaccinated animals 6 weeks after single immunization. Groups of five male Syrian hamsters were vaccinated with PBS or 10^9^ vp of SC-Ad-Spike by the IN or IM route. Six weeks later, they were challenged intranasally with SARS-CoV-2 2020 WA. (**A**) Changes in body weight after challenge. Comparisons are by two-way ANOVA. (**B**) SARS-CoV-2 viral loads in lungs 7 days after challenge determined by qRT-PCR. (**C**) Lung alveolar space on day 7 after challenge calculated in MATLAB from images provided in fig. S5. Comparisons are by one-way ANOVA. Error bars represent SDs (*P < 0.05, **P < 0.01, ***P < 0.001, and ****P < 0.0001).

Given the better long-term protection against SARS-CoV-2 disease by SC-Ads versus RD-Ads, we used SC-Ad to examine the effects of the route of immunization on protection against SARS-CoV-2 soon after vaccination. Groups of five male hamsters were immunized with phosphate-buffered saline (PBS) or 10^9^ vp of SC-Ad-Spike by the IN or IM route and then were challenged 6 weeks later (Fig. 7). SC-Ad-Spike again protected hamsters with less weight loss occurring than in the longer-term study. SC-Ad–immunized animals suffered a slight drop in body weight on day 2 but then rebounded over the subsequent days (Fig. 7A). In contrast, the body weights of PBS control animals fell over the 7-day period. SC-Ad-Spike by the IN route mediated higher body weights than the PBS group on days 3 through 6 (*P* < 0.01 to 0.0001 by two-way ANOVA). SC-Ad-Spike by the IM route protected body weight better than PBS on days 4 through 6 (*P* < 0.05 to 0.0001). Hamsters immunized with SC-Ad-Spike by the IN route had significantly higher body weights on days 4 and 5 than animals that were immunized by the IM route (*P* < 0.05 and *P* < 0.01 on days 4 and 5 by two-way ANOVA).

SARS-CoV-2 viral titers in the lungs were markedly reduced in the SC-Ad–vaccinated animals, with one hamster having no detectable virus (Fig. 7B) although scatter in viral genome levels in the PBS group prevented these from reaching statistical significance by ANOVA. Lung H&E staining of lungs 7 days after challenge revealed that substantial damage occurred in PBS control animals. In contrast, lung structures and alveolar spaces were preserved better in both SC-Ad-Spike–immunized animals with no differences between the routes (Fig. 7C and fig. S5).

### The effects of the route of immunization on the generation of mucosal immune responses in the lung

The data in hamsters showed clearly that SC-Ads mediated stronger immune responses and protection against SARS-CoV-2 than RD-Ad vectors. However, the effects of the route of immunization on protection were less clear in the hamsters in part due to the dearth of immunological reagents against many hamster immune proteins. Given this, we examined the role of mucosal and systemic immunization in mouse models where more reagents are available.

Groups of 10 BALB/c mice were immunized with PBS, SC-Ad expressing Zika E protein by the IN route, or SC-Ad-Spike by either the IN or IM route, and antibody responses were evaluated. It should be noted that this human SC-Ad vector will not replicate efficiently in mice but can still express the spike protein.

Mice immunized with SC-Ad-Spike generated robust immune responses in the systemic compartment as evidenced by the production of high IgG levels in sera and the generation of spike S1–specific interferon-γ (IFN-γ), interleukin-17 (IL-17), and IL-4 T cell responses in splenocytes as assessed by enzyme-linked immunospot (ELISpot) assay (figs. S7 and S8). Bronchoalveolar lavages (BALs) were collected 8 weeks after single immunization to examine how the routes of immunization affected mucosal responses. Both routes of SC-Ad-Spike immunization generated significant IgG antibodies and RBD neutralizing antibodies (fig. S9A). However, only the IN route of vaccination generated IgA antibodies in BALs. None were produced by IM immunization.

BAL samples were also examined for the presence of T cells in the lung from the immunized mice. However, the small number of cells obtained and an absence of known spike T cell epitopes for BALB/c mice precluded testing for spike-specific responses. Flow cytometry was therefore performed instead on BAL cells (fig. S10). No significant increases in CD4 or CD8 T cells were observed in BAL samples after IM immunization by flow cytometry. In contrast, CD8^+^ IFN-γ^+^, CD4^+^ IFN-γ^+^, and CD4^+^ IL-4^+^ T cells were significantly increased in the BALs of IN SC-Ad–immunized animals, but not of IM-immunized animals. Increased CD4 and CD8 T cells were highest in SC-Ad-Spike IN–immunized animals but were still elevated in those immunized intranasally with the control SC-Ad-Zika E vaccine. This suggests that the SC-Ad vaccine platform itself may play an adjuvant role in recruiting responses at mucosal sites when delivered by a mucosal route. These data demonstrate that mucosal vaccination mediates significant increases in the quantity of certain humoral and cellular immune correlates in mucosa and in the lungs when compared to the traditional IM route of immunization.

## DISCUSSION

This study set out to test the utility of antigen gene replication in a vaccine and the effects of the route of immunization against SARS-CoV-2. This work shows that replicating SARS-CoV-2 spike gene amplifies antigen production, amplifies immune responses, and mediates superior protection against SARS-CoV-2 challenge in an animal model that is permissive for both SARS and human Ad. This study also shows that mucosal and systemic immunization are similar in conferring protection against respiratory challenge with SARS-CoV-2 with only subtle benefits provided by IN immunization.

SC-Ad expressed higher levels of SARS-CoV-2 spike protein than the matched RD-Ad vector. This is consistent with previous comparisons (*2*, *3*, *13*). The ability to replicate translated into 100-fold higher antigen expression in human cells. This higher expression by SC-Ad-Spike translated into higher and more persistent serum antibody responses than RD-Ad-Spike in semi-Ad–permissive Syrian hamsters after single immunization by either the IM or IN route. This higher antigen expression translated into better protection against SARS-CoV-2 challenge by SC-Ad by either the IN or the IM route when compared to RD-Ad. Notably, good protection was observed more than 10 months after a single immunization. This contrasts to other vaccine platforms that may require two, three, or more rounds of vaccination (*17*).

While replicating vaccines are documented to be more robust than nonreplicating vaccines (*3*, *13*, *18*), they also ideally have some safety switch to ensure that they themselves do not cause their own infections. SC-Ad’s safety switch is the deletion of a pivotal viral cement protein gene that renders the vaccine unable to produce infectious progeny viruses. While SC-Ad does not generate progeny viruses and has better safety than a fully replicating virus, any cell that is infected by SC-Ad will die due to the Ad life cycle just like an RC-Ad. While this is true, it is also true that any cell infected by any RD-, SC-, or RC-Ad will also ultimately be killed by anti-Ad T cells. Likewise, any good SARS-CoV-2 vaccine that generates anti-spike T cells will also lead to the destruction of transduced or transfected cells that express the spike protein. This is true for RD- and SC-Ad vaccines but is also true for mRNA or other platforms that deliver antigens into host cells.

Despite the biology that will drive direct cell death through the Ad life cycle and antiviral or antivaccine T cell responses, SC-Ad vaccines appear to be relatively safe as demonstrated in hamsters given IN or IM doses of 10^11^ vp of SC-Ad expressing *Clostridium difficile* vaccine (*19*) and by the fact that SC-Ad-Spike passed sufficient safety tests to enter phase 1 clinical trials (NCT04839042).

The higher potency per vaccine particle of SC-Ad versus RD-Ad can allow one to use the same number of vaccine particles as RD-Ad to generate stronger protection in a single person. Alternately, one can harness SC-Ad potency by reducing the number of particles delivered per person to equal RD-Ad potency but stretch each batch of vaccine 100 times to perhaps vaccinate 100 times more people. This could be pivotal for deploying vaccines globally for the next pandemic.

Given where we are in the pandemic, with many already vaccinated with mRNA or RD-Ad vaccines, SC-Ad-Spike might best be deployed as an IN booster to amplify mucosal protection in humans who have already received their mRNA, Ad26, and ChAd vaccines by the IM route.

The levels of antibodies in sera were higher in animals immunized by the IM route compared to the IN route. These higher sera responses in blood did not directly translate into better protection. IN vaccinated animals were provided equal protection to IM vaccines. In some cases, the IN vaccine provided superior protection against weight loss due to SARS-CoV-2 infection. This may be due to the ability of IN vaccination with the Ad vaccines to increase the recruitment of IgA antibodies and CD4 and CD8 T cells into the lumen of the lung when compared to IM immunization.

While the benefits of IN vaccination were subtle in this study, there may be other merits to this route of mucosal vaccination against SARS-CoV-2 and other pathogens. One benefit is that IN vaccination does not require the use of needles. This means that this route of vaccination does not require sterile needles and does not generate a sharp biohazard with every person that is immunized. This may be critical for global vaccination efforts particularly in regions with less infrastructure to provide sterile needles and control medical biological waste.

It could also be that IN immunization might bypass another significant challenge for Ad vaccines: vaccine-induced thrombotic thrombocytopenia (*20*–*32*). There are data suggesting that Ad26 and ChAdOx1 COVID-19 vaccines may induce this side effect by binding platelet factor 4 (PF4) and driving anti-PF4 antibody responses after leaking into the blood after IM injections (*33*). If this biology plays a role, one simple sidestep of this side effect would be to avoid injecting Ad vaccines at all. Instead, one could deliver the vaccines by the IN route to avoid leaking into the blood and binding PF4.

Whether IN immunization with SC-Ad or other vaccines will have utility in humans against SARS-CoV-2 in humans remains to be determined. Regardless, this study demonstrates that replicating SC-Ad vaccines have significantly more potency against SARS-CoV-2 than RD-Ad vaccines. This work with Ad gene-based vaccines provides proof of principle for the utility of replication to amplify protection by gene-based vaccines against SARS-CoV-2 and other infectious diseases.

## METHODS

### SC-Ad expressing wild-type SARS-CoV-2 spike

A codon-optimized cDNA encoding the original wild-type spike protein from SARS-CoV-2 isolate 2019-nCoV_HKU-SZ-002a_2020, accession number MN938384.1, was synthesized by Genewiz. This full-length sequence was inserted into the shuttle plasmid pAd6-NdePfl-CMV-MCS-3X-LZL. This sequence was recombined into pAd6-ΔE1-ΔE3 and pAd6-ΔIIIa-ΔE3 by red recombination as in (*2*–*4*) to generate RD-Ad-spike and SC-Ad-Spike, respectively. These viruses were cut with AsiSI to liberate their viral genomes, and these were transfected into 293-IIIA cells to rescue the viruses. The viruses were purified from 10 Plate CellStacks (Corning) on two CsCl gradients and used as vp based on OD_260_ (optical density at 260 nm) measurements (*2*–*4*).

### Western blotting

Human A549 lung cells were infected with RD-Ad-Spike or SC-Ad-Spike at the indicated MOIs and harvested 24 hours later. A Western blot was performed against the samples using SARS-CoV-2 spike mouse monoclonal antibody [1A9] (GeneTex), diluted in blocking buffer at a 1:1000 dilution followed by a horseradish peroxidase (HRP) secondary antibody (Invitrogen) at a 1:10,000 dilution. HRP was detected using SuperSignal West Maximum Sensitivity Substrate (Thermo Fisher Scientific) and imaged on the ChemiDoc Imaging System (Bio-Rad).

### Animals

BALB/c mice were purchased from Charles River Laboratories. Syrian hamsters were purchased from Envigo. Mice and hamsters were housed in the Mayo Clinic Animal Facility and in the University of Texas Medical Branch (UTMB) Animal Facility. All animal handling and experiments were carried out according to the provisions of the Animal Welfare Act, Public Health Service Animal Welfare Policy, the principles of the National Institutes of Health’s *Guide for the Care and Use of Laboratory Animals*, and the policies and procedures of the Mayo Clinic and the UTMB. This study was conducted in Mayo Clinic’s Association for the Assessment and Accreditation of Laboratory Animal Care–accredited facilities and was approved by the Institutional Animal Care and Use Committee. For hamster studies, each vaccine was blinded to the personnel administering them to the animals, and subsequent assays were performed by the same person in a blinded fashion. The group key was provided to M.A.B. at time of data analysis. The male hamsters that were challenged with SARS-CoV-2 10 months after single immunization were shipped from Mayo Clinic to UTMB as blinded groups. These groups were not unblinded until the end of the challenge study.

### Immunizations

Mice and hamsters were anesthetized with isoflurane before immunizations by the indicated routes: IM or IN. For IM immunization, 50 μl of a solution of virus diluted in PBS was injected into each flank for a total volume of 100 μl per animal. IN immunization was performed by pipetting 40 μl of a solution of virus diluted in PBS dropwise into the nostrils of each animal. Each animal received a total volume of 40 μl, alternating pipetting between nostrils.

### Bronchoalveolar lavage

BALs were performed as described in (*34*). Mice were euthanized via CO_2_ gas and then sterilized with 70% ethanol. Scissors were used to open the chest cavity up to the chin and to expose the trachea. A razor was used to puncture the trachea, and 1 ml of PBS was pipetted into and out of the lungs. This was repeated two additional times, giving a total volume of 3 ml. Cells were then pelleted out of the BAL fluid by centrifugation. The cells were used to run flow cytometry, and the supernatant fluid was used to run ELISA.

### Lung tissue single-cell suspension

Lung cells were isolated as described in (*35*). Briefly, lungs were extracted and processed with a gentleMACS dissociator (Miltenyi Biotec) and placed in gentleMACS C tubes containing 2.5 ml of RPMI 1640, 40.4 μl of concentration Roche Liberase TM (14 U/ml), and 62.5 μl of deoxyribonuclease I at a concentration of 1 mg/ml. Program Lung_01 was performed on the gentleMACS dissociator, followed by a 1-hour incubation at 37°C. RPMI 1640 (2.5 ml) with 10% fetal bovine serum (FBS) was added before running program Lung_02. All tubes were centrifuged for 5 min at 250*g*, and the contents were transferred to a 50-ml conical tube via a 70-μm mesh. RPMI 1640 (2.5 ml) with 10% FBS was added to the gentleMACS tubes and poured over the mesh to wash. Conical tubes (50 ml) were centrifuged for 10 min at 250*g*, and the supernatant was aspirated and discarded. Cell pellets were resuspended in 2 ml of ammonium-chloride-potassium lysis buffer and centrifuged for 5 min at 350*g*. Supernatants from this reaction were discarded, and the cell pellet was washed by resuspending in 2 ml of PBS and was centrifuged for 5 min at 350*g*. Supernatants were extracted and discarded. The cell pellet was resuspended in desired volume RPMI 1640 and analyzed by flow cytometry.

### Sample collections

At indicated time points, the animals were anesthetized with isoflurane, and serum was collected by cheek bleed in mice or from jugular veins in hamsters. In addition, for BAL, the mice were euthanized via CO_2_, and BAL was performed according to the procedure described.

### Antibody ELISAs

Binding IgG and IgA antibody responses in mouse serum, hamster serum, and BAL fluid were measured by ELISA against spike S1 protein and SARS-CoV-2 RBD variants. Flat-bottom plates (Thermo Fisher Scientific) were coated with spike S1 antigen at 10 ng per well in 100 μl of PBS or with SARS-CoV-2 RBD variant antigen at 100 ng per well in 100 μl of PBS, including a triplicate of negative control wells, which received no protein antigen. The protein antigen used for most SARS-CoV-2 ELISAs was recombinant SARS-CoV-2 (2019-nCoV) spike S1 fused to Ig heavy chain (S1-Fc) from Sino Biological. The protein antigen used for the SARS-CoV-2 variants included S1 proteins and RBD recombinant proteins, also from Sino Biological. These included His6-tagged RBD proteins expressed from 293 human cells (wild-type; K417N; N439K; Y453F; S477N; E484K; N501Y; and K417N, E484K, N501Y) and a triple mutant corresponding to the beta variant (B.1.351). Two spike S1 proteins were also tested that had a single D614G mutation or combined K417N, E484K, N501Y and D614G mutations.

Plates were left overnight at 4°C. Plates were washed two times with 200 μl of 1× tris-buffered saline with 0.1% Tween-20 (TBST), followed by adding 200 μl per well of blocking buffer, consisting of 5% milk powder in TBST, for 2 hours at room temperature. Plates were washed two times with 200 μl of 1× TBST. All samples were run in triplicate, including a triplicate of positive and negative control wells in each plate. Samples were serially diluted in blocking buffer and were transferred to the assay plate and incubated for 3 hours at room temperature. For the positive control wells, SARS-CoV-2 spike antibody [1A9] (GeneTex) was used as the primary antibody. Plates were washed four times with 200 μl of 1× TBST, followed by the addition of the secondary antibody. For hamster samples, the secondary antibodies used were Peroxidase Conjugated Affinity Purified Anti-Golden Syrian Hamster IgG (H&L) Goat (Rockland Inc.) and Rabbit Anti-Hamster IgA (Brookwood Biomedical). For mouse samples, the secondary antibodies used were GOXMO HRP HIGH XADS (Invitrogen) and HRP-Goat Anti-Mouse IgA (Invitrogen). The secondary antibody for the positive controls was Purified Recomb Protein A/G Peroxidase Conjugated (Invitrogen). Plates were left to incubate with the primary antibody for 2 hours at room temperature. Plates were washed four times with 200 μl 1× TBST. 1-Step Ultra TMB-ELISA (50 μl) was added to each well and left at room temperature for 30 min, and then, 50 μl of 2 M sulfuric acid was added to each well. Plates were read at 450 nm in a Synergy H1 microplate reader (BioTek). All statistical analyses were done by one-way ANOVA.

### SARS-CoV-2 neutralization assays

RBD pseudo-neutralization assays were performed on hamster serum using the cPass Neutralization Antibody Detection kit (GenScript). This assay involves mixing test sera with an epitope-tagged spike RBD domain corresponding to the original virus sequence. These mixtures are then applied to an ELISA plate coated with ACE2. If there is no RBD neutralization, then RBD binds ACE2, and its tag is detected in ELISA. If the sample inhibits RBD binding to ACE2, the tag signal is reduced. The plate OD is read, and RBD neutralization rate is calculated using the mathematical formula provided with the kit.

SARS-CoV-2 plaque reduction neutralization test (PRNT) assays were performed in the Galveston National Laboratory at the UTMB. Procedures involving live virus were conducted under biosafety level 3 conditions. Test samples for the PRNT assay were removed from frozen storage (−80°C), thawed under ambient conditions, and then heat-inactivated in a 56°C water bath for 60 min. SARS-CoV-2 test inoculum was prepared fresh from frozen stock in minimum essential medium (MEM; Gibco) to a final concentration of 1000 plaque-forming units/ml. Test and control samples were serially diluted twofold in MEM and 2% heat-inactivated FBS beginning at a 1:10 dilution. An equivalent volume of SARS-CoV-2 inoculum was added and incubated for 60 min at 37°C. Samples were added to confluent Vero 76 cell monolayers in 96-well format incubated at 37°C/5% CO_2_ for 48 hours. Following incubation, each well was inspected via microscope for cytopathic effects (CPEs). Wells that contained no CPE were recorded as positive for antibody neutralization, and wells with CPE were recorded as negative for antibody neutralization. The PRNT_50_, defined as the reciprocal of the highest dilution resulting in 50% plaque reduction compared to virus-only control, was determined for each sample. The limit of detection was defined as one plaque per well. The LLOQ was 20.

### SARS-CoV-2 challenge

These studies were performed at UTMB under Biosafety Level 3 conditions. Groups of five Syrian hamsters were vaccinated as indicated and administered 10^6^ TCID_50_ SARS-CoV-2 (USA_WA1/2020) intranasally with 50 μl per nare as in (*35*). The hamsters were monitored and scored daily for alterations in activity, behavior, appearance, and body weight. Body weights were normalized to each animal’s starting weight. Nasal samples were collected on days 1, 3, and 5 using 0.5-mm Microbrush Applicators to swab the inner surfaces of the nares and were placed into sterile PBS (0.5 ml).

Seven days after challenge (or upon meeting endpoint criteria), the hamsters were humanely euthanized by carbon dioxide inhalation followed by bilateral thoracotomy. The lungs from each hamster were removed, weighed, and examined for SARS-CoV-2 viral loads and histopathology. For viral loads, half of the left lobe of the lung was homogenized and centrifuged, and the resulting supernatant was analyzed for infectious virus via TCID_50_ assay and for viral RNA by qRT-PCR.

### SARS-CoV-2 qRT-PCR

qRT-PCR was performed against the SARS-CoV-2 E gene as in (*36*). Nasal swab suspension samples (50 μl) were added to TRIzol LS Reagent (250 μl) and allowed to incubate under ambient conditions for 10 min. Samples were processed to RNA using Zymo Direct-zol RNA Mini Prep kits (Zymo Research, Irvine, CA, USA) per the manufacturer’s instructions. RNA samples were analyzed via qRT-PCR targeting the SARS-CoV-2 E gene to detect genomic RNA, not subgenomic RNAs. The probe (Integrated DNA Technologies, Coralville, IA, USA) was labeled at the 5′-end with fluorophore 9-carboxyfluorescein (6-FAM) and included an internal quencher (ZEN) and a 3′-end quencher (IowaBlackFQ, IABkFQ). The master mix was prepared by combining forward primer (250 nM, 5′-ACAGGTACGTTAATAGTTAATAGCGT-3′), reverse primer (250 nM, 5′-ATATTGCAGCAGTACGCACACA-3′), and probe (375 nM, 5′-6FAM-ACACTAGCC/ZEN/ATCCTTACTGCGCTTCG-IABkFQ-3′) with 12.5 μl of 2× QuantiFast Probe Mix (QIAGEN, Germantown, MD, USA), 0.25 μl of 2× QuantiFast RT Mix (QIAGEN), and PCR-grade water (fill to 20 μl). A test sample (5 μl) was added to the master mix, resulting in a final volume of 25 μl per reaction. Real-time analysis was performed using the Bio-Rad CFX96 Real-Time PCR Detection System. Thermocycling conditions were as follows: step 1, 1 cycle, 50°C for 10 min; step 2, 1 cycle, 95°C for 10 min; steps 3 to 5, 45 cycles, 95°C for 10 s and 60°C for 30 s, single read. Negative controls included reaction mixtures without RNA. For quantification purposes, a serial diluted synthetic oligonucleotide was used to generate the standard curve. All qRT-PCR results are expressed as genome equivalents per gram.

### SARS-CoV-2 viral TCID_50_

Infectious SARS-CoV-2 was measured by TCID_50_ analysis as in (*36*). Samples were serially diluted and incubated on Vero C1008 (E6) cells (BEI Resources, NR-596, Lot 3956593) in 96-well plates with negative and positive control. The plates were incubated for 96 hours when CPE was measured by microscopic observation. The LLOQ for the TCID_50_ assay was 1.47 × 10^2^ TCID_50_/ml. The LLOQ for TCID_50_ was 2.50 × 10^2^ TCID_50_/g of tissue.

### Lung sectioning, staining, and imaging

After sacrifice, hamster lungs were extracted and treated with 10% formalin for more than 30 days. The lungs were incubated in 15% sucrose for 3 hours followed by incubation in 30% sucrose overnight, both at 4°C. Tissues were transferred into optimal cutting temperature medium for 2 hours and then were frozen in isopentane on dry ice. Samples were stored at −80°C until they were sectioned. Tissues were sectioned in 18-μm thicknesses on a cryostat. Slides were stained with H&E (ScyTek Laboratories Inc.) according to the package instructions. Stained slides were imaged via light microscopy at ×20 magnification on an Olympus SC50. Three representative images were collected from each lung as shown in fig. S5. To quantify damage to the lungs, alveolar space was quantified using a custom script in MATLAB (fig. S9). To count “pixels” of open alveoli in the images, regions stained with H&E were eliminated from the image in Photoshop 2022 using the “Color Range” function. In this mode, the Eyedropper Tool was used to select the background color of the alveolar space. The selection was inverted and applied to the image. This process selected all pixels whose color was not identical to that of the alveolar space. These selected pixels were deleted from the image, leaving only the pixels associated with the alveolar space, surrounded by a black background (see fig. S5). This process was repeated for every image. The processed pictures were analyzed by a custom script in MATLAB and to quantify the remaining pixels to represent alveolar space in each image. Values from three representative images were obtained per animal and were used for calculations in graphs.

### ELISPOT assay for detecting antigen-specific IFN-γ–producing cells

Freshly isolated splenocytes were stimulated with spike S1 or S2 subunit protein (1 μg/ml) to determine the numbers of cells producing IFN-γ, IL-4, or IL-17 by the ELIspot assay using the methodology reported previously (*36*). Briefly, splenocytes were plated at 2.5 × 10^5^ cells per well in triplicate in 96-well plates. Cells were incubated at 37°C with medium alone, human papilloma virus E7 peptide (negative control), SARS-CoV-2 spike protein S1 subunit (1 μg/ml), SARS-CoV-2 spike protein S2 subunit (1 μg/ml), tetanus toxoid (negative control, 100 ng/ml), or concanavalin A (positive control, 10 ng/ml). After 24 hours, cells were transferred to nitrocellulose plates, coated with anti–IFN-γ, anti–IL-4, or anti–IL-17 antibody, and incubated for an additional 24 hours. Plates were then washed and incubated with biotinylated anti–IFN-γ, anti–IL-4, or anti–IL-17 antibody, streptavidin-alkaline phosphatase, and colorimetric substrate, with washes between each step. After drying overnight, the plates were read on an AID ELIspot reader (San Diego, CA). Antigen-specific T cells were defined as the average number of spots elicited by the antigen of interest minus the average number of spots elicited when cells were incubated with culture medium alone, without the addition of any peptides.

### Flow cytometry

BAL samples were centrifuged at 1500 rpm for 5 min, and the supernatant BAL fluid was removed. The cell pellet was resuspended in 200 μl of T Cell Media [Iscove’s modified Dulbecco’s medium (IMDM) with 10% FBS, penicillin/streptomycin, and 2-mercaptoethanol (2-ME)] and was then added to a 96-well plate. Two microliters of stimulating mix (ionomycin, 500 μg/ml; phorbol 12-myristate 13-acetate, 50 μg/ml; and 445 μl of T Cell Media) was added to each well to be stimulated and was incubated overnight at 37°C. One hundred microliters of a GolgiPlug Mix (1 μl of GolgiPlug/1 ml of T Cell Media) was added to each well and thoroughly mixed by pipetting before incubating for 4 to 6 hours at 37°C. Cells were centrifuged at 1500 rpm for 5 min, and the supernatant was removed. Cell surface antibodies were added in 50-μl total volume in fluorescence-activated cell sorting (FACS) buffer and then incubated 30 min on ice. Plates were washed twice with FACS buffer, resuspending cells in FACS buffer and centrifuging for 5 min at 1500 rpm to remove the supernatant. The cells were then pelleted by centrifugation at 1500 rpm for 5 min. Cell pellet was resuspended in 100 μl of the Fix/Perm Solution for 20 min at 4°C before being washed twice with 1× BD Perm/Wash Buffer and then centrifuging at 1500 rpm for 5 min to pellet. Intracellular cytokine antibodies were diluted in 50 μl of 1× BD Perm/Wash Buffer and added to each well. Plates were then incubated 30 min on ice. Plates were washed twice with 1× BD Perm/Wash Buffer, resuspending cells in 1× BD Perm/Wash Buffer and centrifuging for 5 min at 1500 rpm to remove the supernatant. Cells were resuspended in 500 μl of 1% paraformaldehyde and left at 4°C overnight before flow cytometry analysis.

### Statistical analysis

Prism 9 GraphPad software was used for all statistical analyses.

## Notes

View/request a protocol for this paper from *Bio-protocol*.
