# Supplementary material for: Comparison of replicating and nonreplicating vaccines against SARS-CoV-2
Source: Sci Adv. 2022 Aug 24;8(34):eabm8563. doi: 10.1126/sciadv.abm8563 (PMC9401629; doi:10.1126/sciadv.abm8563)
Supplement: Supplementary file 1 — Figs. S1 to S10 [file sciadv.abm8563_sm.pdf]

Science Advances
AAAS

Supplementary Materials for

Comparison of replicating and nonreplicating vaccines against SARS-CoV-2

Haley E. Mudrick *et al.*

Corresponding author: Michael A. Barry, mab@mayo.edu

Sci. Adv. **8**, eabm8563 (2022)
DOI: 10.1126/sciadv.abm8563

This PDF file includes:

Figs. S1 to S10

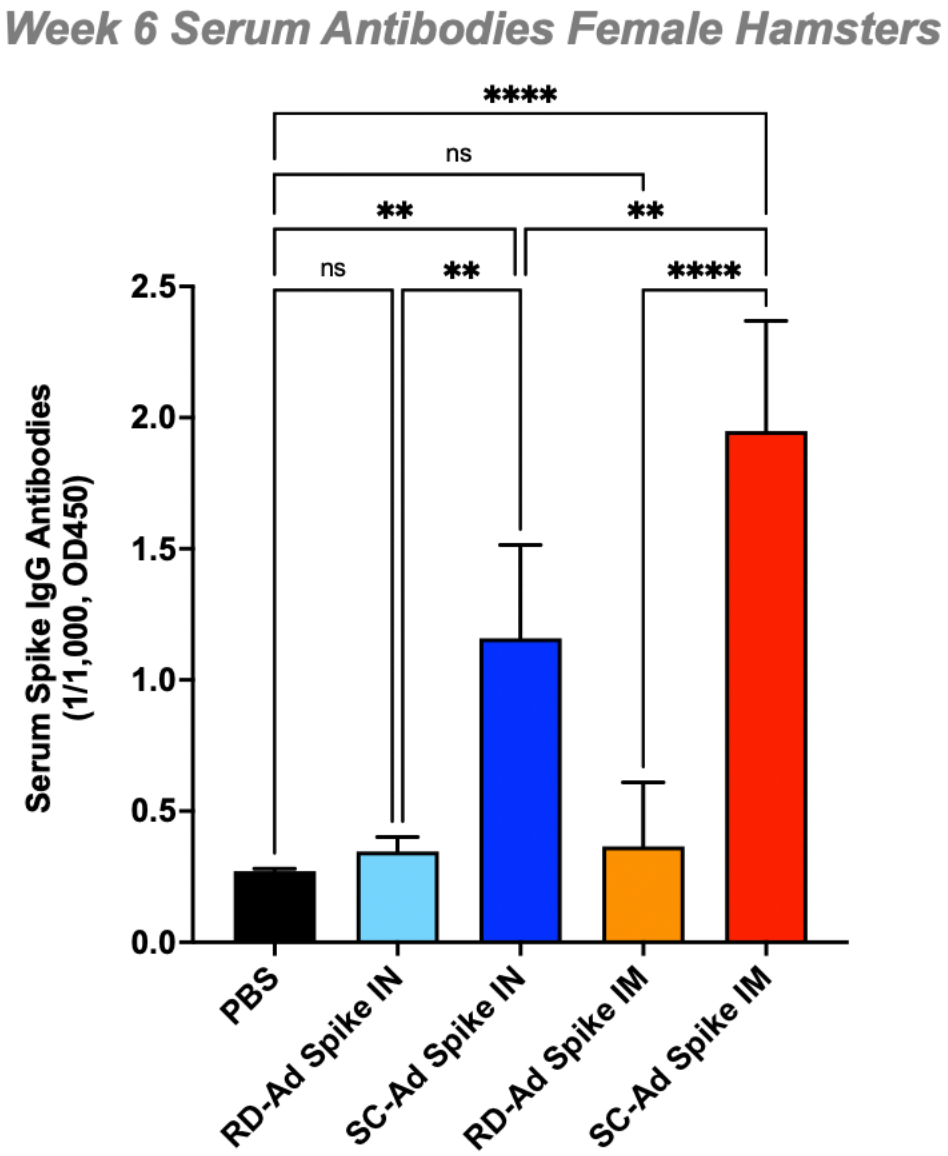

Supplemental Fig. 1. Spike Antibody Production by RD-Ad and SC-Ad Vaccines after a Single Intranasal or Intramuscular Vaccination in Female Hamsters. Female Syrian hamsters were immunized at a dose of 10^9 vp, and sera were collected 6 weeks after immunization were and tested for spike antibodies by ELISA. Error bars represent standard deviations. (**** = p<0.0001, *** = p<0.001, ** = p<0.01, * = p<0.05 by one way ANOVA)

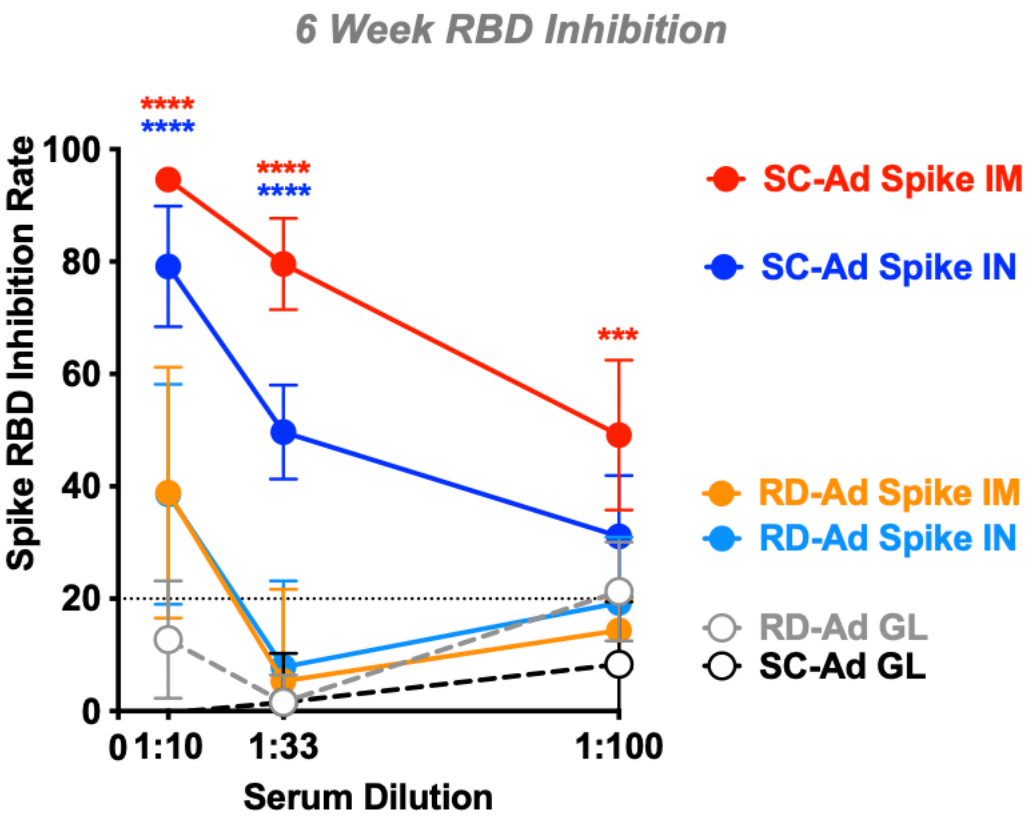

Supplemental Fig. 2. SARS-CoV-2 Spike RBD neutralization. RBD neutralization was performed at 1:10, 1:33, and 1:100 dilutions of week 6 serum from male hamsters comparing RD-Ad spike and SC-Ad spike at intranasal and intramuscular routes of immunization. Spike inhibition rate was determined based on the formula provided by Genscript. Error bars represent standard deviations. (**** = p<0.0001, *** = p<0.001, ** = p<0.01, * = p<0.05).

Supplemental Figure 3 Mudrick *et. al.*

Week 14 Male Hamster
IM SC-Ad-GL and SC-Ad-Spike Groups

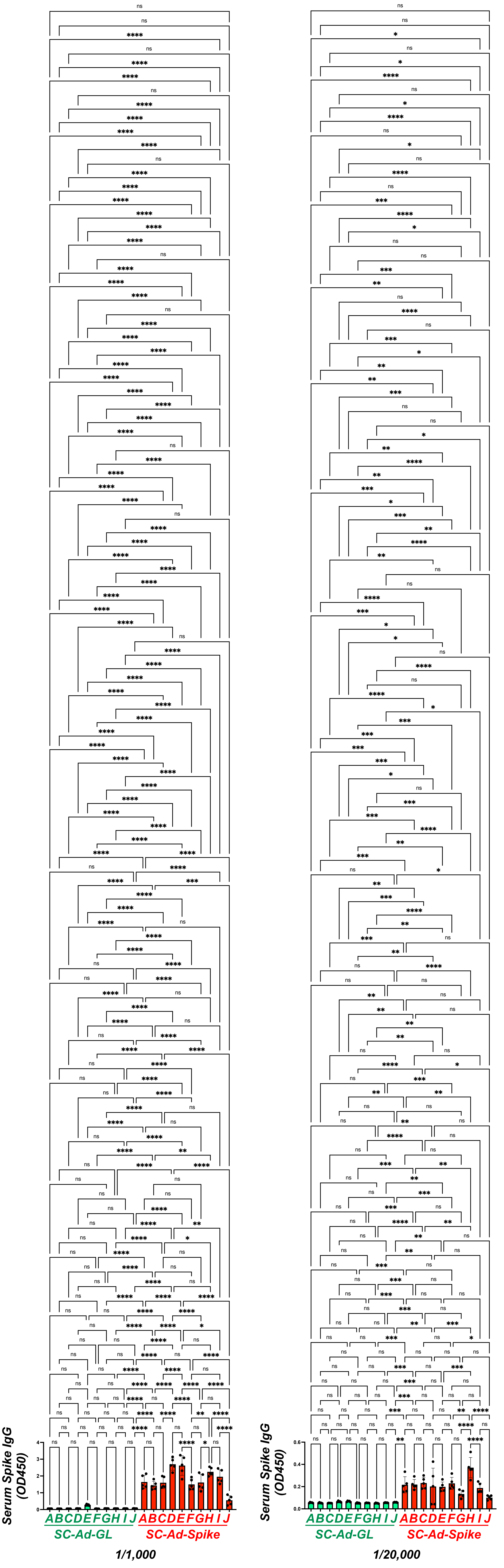

A: *K417N RBD*
B: *N439K RBD*
C: *Y453F RBD*
D: *S477N RBD*
E: *E484K RBD*
F: *N501Y RBD*
G: *K417N, E484K, N501Y RBD*
H: *D614G S1*
I: *K417N, E484K, N501Y, D614G S1*
J: *SARS-1 RBD*

Supplemental Fig. 3. ANOVA Comparisons of RD-Ad and SC-Ad Vaccines after a Single Intranasal or Intramuscular Vaccination in Male Hamsters. Data from Figure 2. Error bars represent standard deviations. (**** = p<0.0001, *** = p<0.001, ** = p<0.01, * = p<0.05).

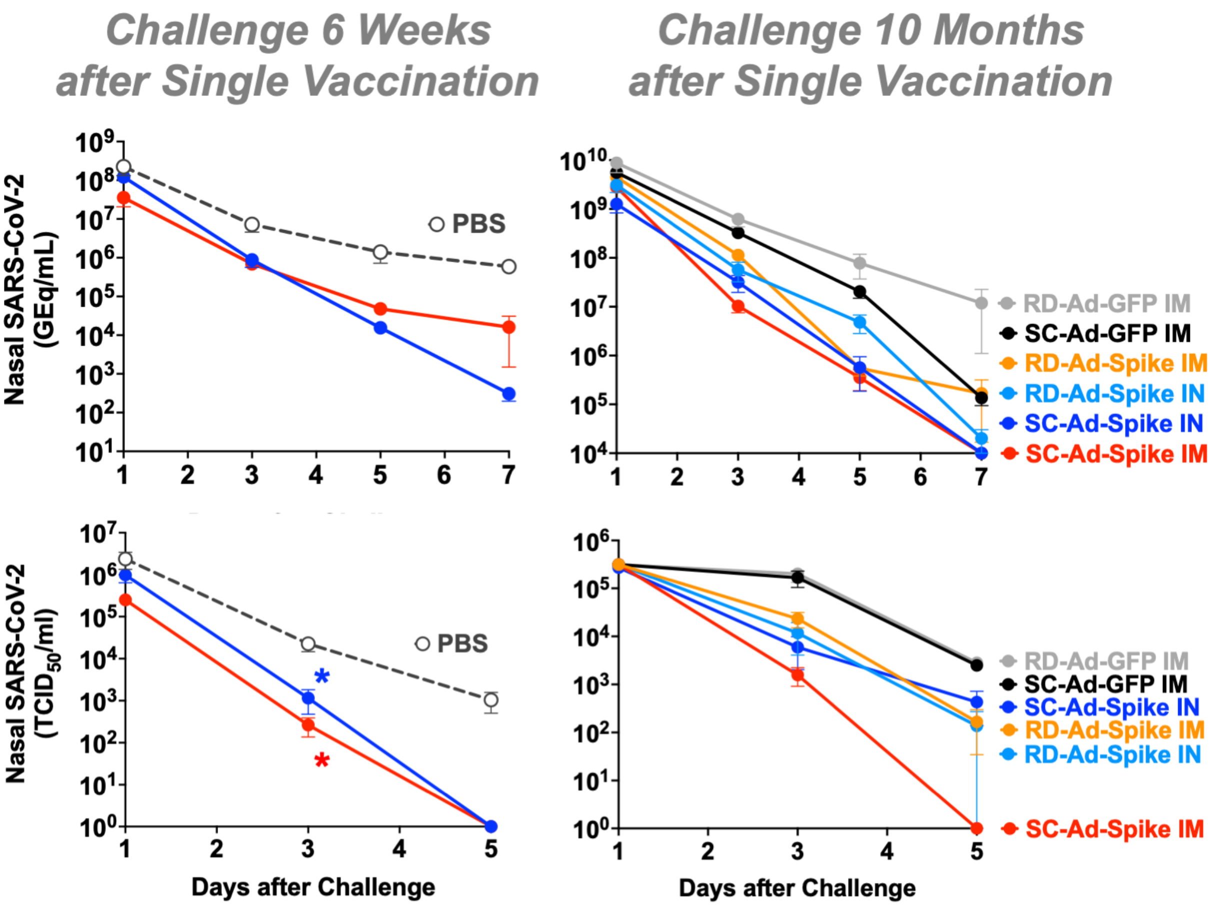

Supplemental Fig. 4. Nasal SARS-CoV-2 Viral Loads after Challenge. Data is shown for days after intranasal challenge at 6 weeks or 10 months after single immunization in terms of q-RT-PCR and by $TCID_{50}$ assay. Error bars represent standard deviations. * $p < 0.05$ by one way ANOVA at that time point.

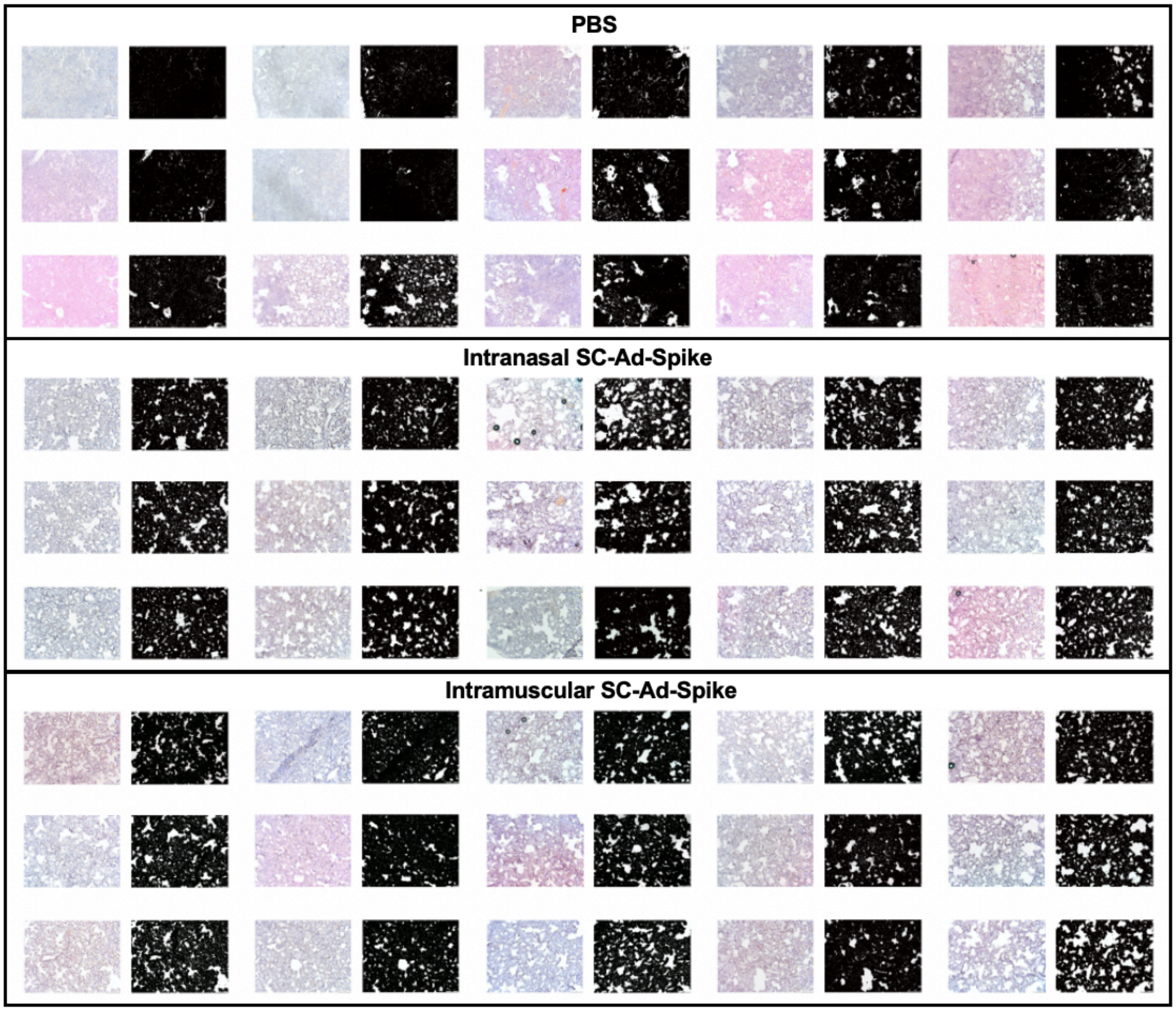

Supplemental Fig. 5. Lung Sections from Animals Challenged with SARS-CoV-2 6 Weeks after Single Immunization. Lungs were collected 7 days after challenge. They were sectioned and stained with H&E (Left Panels of Each). Each image was digitized (Right Panels) and the number of alveoli space pixels from 15 images per group were quantified using MatLab as in **Supplemental Figure 6** to produce **Figure 7C**.

Alveoli H&E Analysis

Initialize

```
clear all
close all
clc
% Read in image
holder = imread('Lung 2.png');
```

Segment Image

binarize

```
BW = imbinarize(rgb2gray(holder), 'adaptive');
% show binary image
figure, imshow(BW), title('Binarized H&E Image')

hblob = vision.BlobAnalysis( ...
            'AreaOutputPort', true, ...
            'BoundingBoxOutputPort', false, ...
            'OutputDataType', 'single', ...
            'MinimumBlobArea', 1, ...
            'MaximumBlobArea', 30000, ...
            'MaximumCount', 150000,...
            'PerimeterOutputPort', true);
% Calculate airspace area vector
[area] = hblob(BW);
% Sum airspace area vector for total alveolar area
total_area = sum(area)
% Define alveolar perimeter
perimeter = bwperim(BW);
% Sum alveolar perimeter vector for total alveolar perimeter
total_perimeter = sum(perimeter(:) == 1)
% Print figure of H&E image, alveolar space, and alveolar perimeter
figure()
subplot(2,2,1), imshow(holder), title('H&E Image')
subplot(2,2,2), imshow(BW), title('Alveolar Area')
subplot(2,2,3), imshow(perimeter), title('Alveolar Perimeter')
```

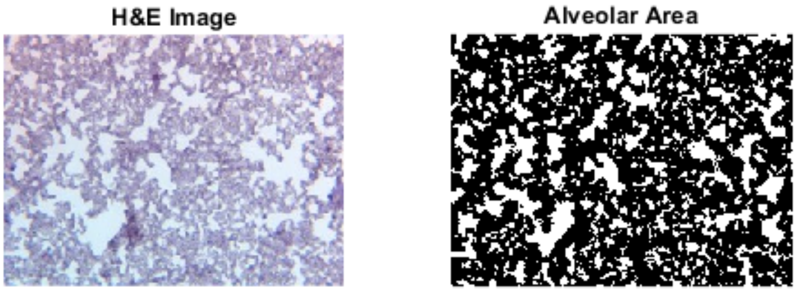

Supplemental Fig. 6. Matlab Analysis of H&E Stained Lungs. A) Matlab script *Published with MATLAB® R2020a*. **B)** Representative conversion of H&E to alveolar area.

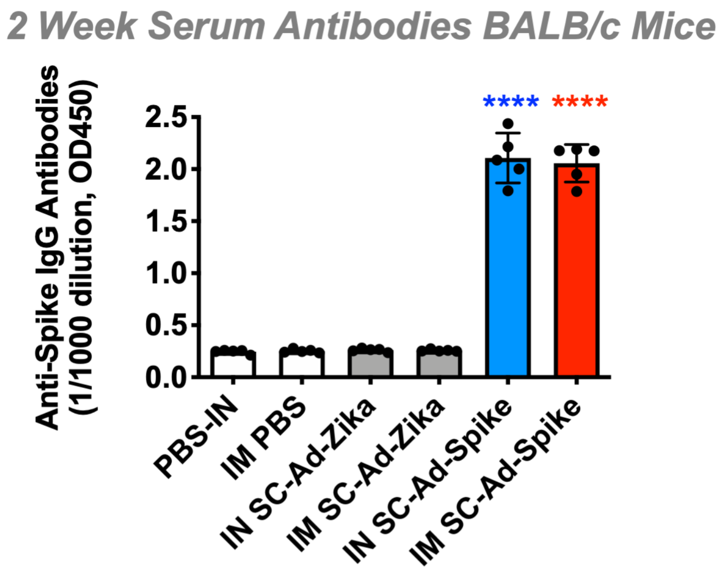

Supplemental Fig. 7. Serum Antibody Production to Spike Protein after Single Intranasal or Intramuscular Administration of SC-Ad-Spike in Mice. Male BALB/c mice were immunized at a dose of 10^{10} vp virus, and serum was collected 2 weeks after single immunization and was tested for SARS-CoV-2 spike IgG antibodies by ELISA. Error bars represent standard deviations. (**** = $p<0.0001$, *** = $p<0.001$, ** = $p<0.01$, * = $p<0.05$)

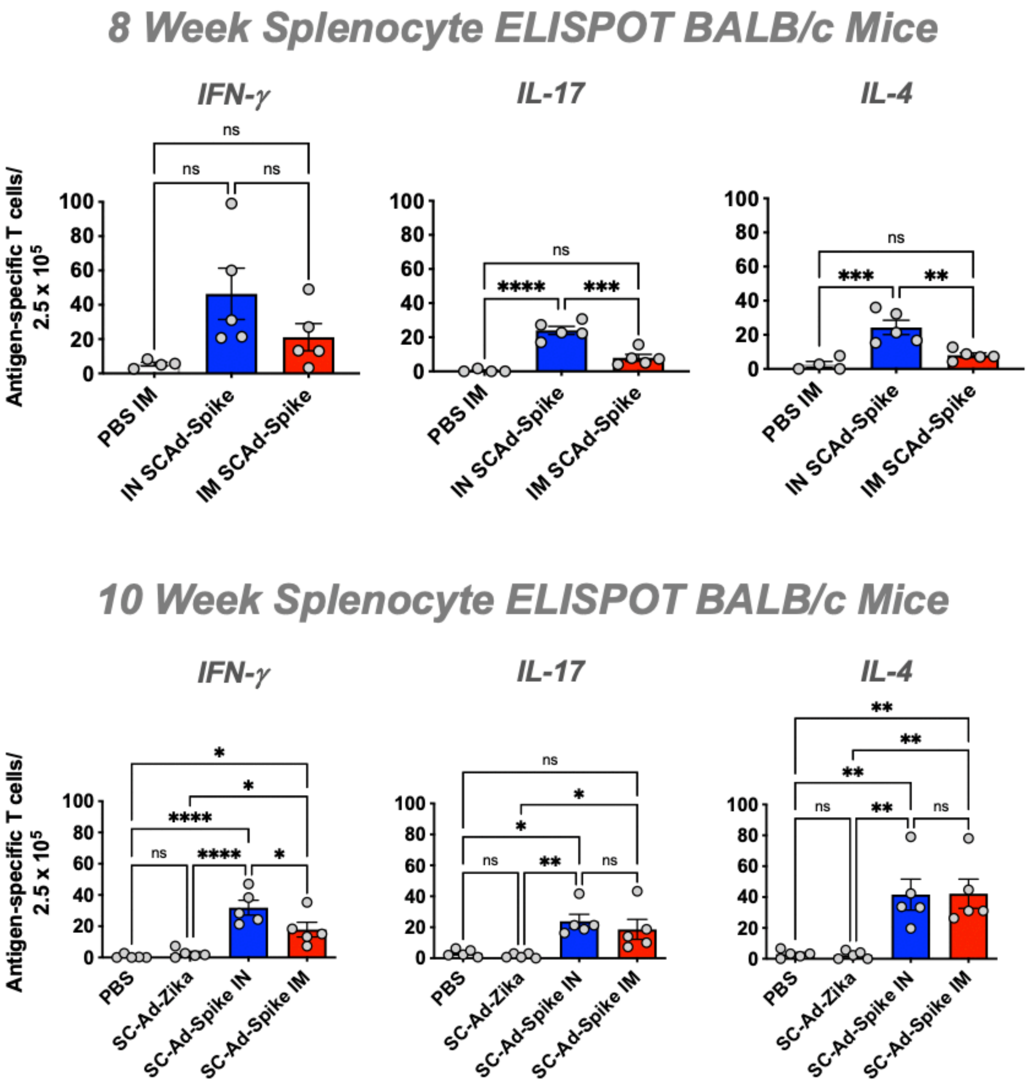

Supplemental Fig. 8. ELIspot Responses in Splenocytes after a Single Intranasal or Intramuscular Vaccination in Mice. BALB/c mice were immunized by the indicated routes with 10^{10} vp of the indicated SC-Ad vectors or PBS. Splenocytes were stimulated with whole S1 spike subunit and IFN-γ, IL-4, or IL-17 ELIspot assays were performed. One half of mice from the SC-Ad-Spike and PBS groups were tested at 8 weeks. The other half were tested 10 weeks after immunization along with SC-Ad-Zika control mice. Error bars represent standard deviations. (**** = $p<0.0001$, *** = $p<0.001$, ** = $p<0.01$, * = $p<0.05$)

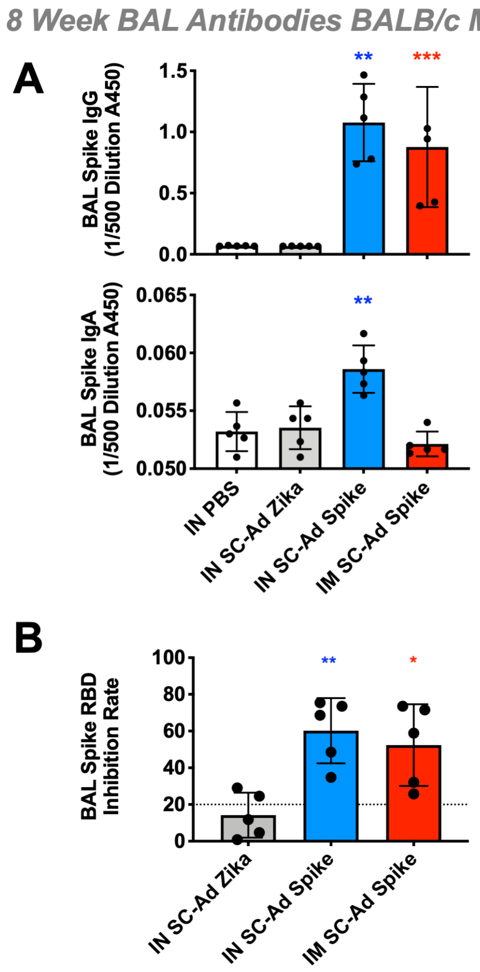

Supplemental Fig. 9. **Mucosal Antibody Production to Spike Protein in Bronchoalveolar Lavages (BALs) after Single Intranasal or Intramuscular Administration of SC-Ad-Spike in Mice.** Male BALB/c mice were immunized with 10^{10} vp of SC-Ad-Spike and bronchoalveolar lavage (BAL) fluid was collected at 8 weeks after single immunization. **A)** BAL fluid was used at 1:500 dilution to test for SARS-CoV-2 spike IgG and IgA antibodies by ELISA. Plates were read at 450nm, and all analyses were done by one-way ANOVA. **B)** SARS-CoV-2 neutralization assay (Genscript) was performed at 1:10 dilution of BAL fluid, comparing IN SC-Ad Zika, IN SC-Ad spike and IM SC-Ad spike. Error bars represent standard deviations. (*** = $p<0.001$, ** = $p<0.01$, * = $p<0.05$).

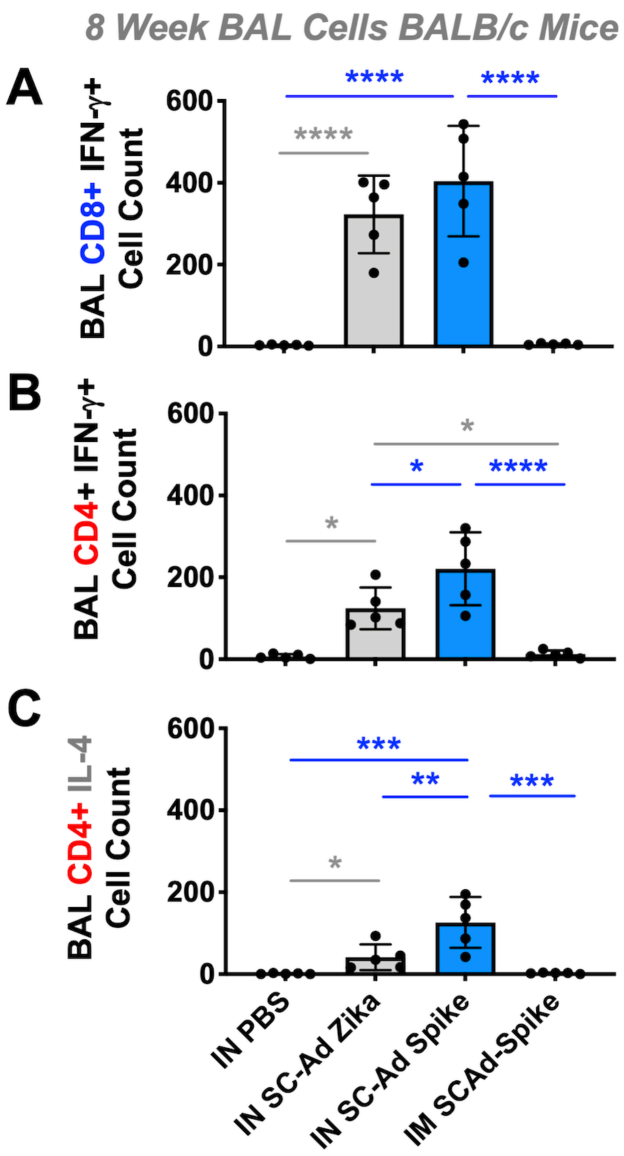

Supplemental Fig. 10. Changes in T Cell Populations in Bronchoalveolar Lavages (BALs) after Single Intranasal or Intramuscular Administration of SC-Ad-Spike in Mice. 8 weeks after single immunization of 10^{10} vp of SC-Ad-Spike in BALB/c mice, BAL was performed. Cells were pelleted out of BAL fluid and analyzed by flow cytometry. **A)** Shows number of CD8 T cells counted that expressed IFN-γ. **B)** Shows number of CD4 T cells counted that expressed IFN-γ. **C)** Shows number of CD4 T cells counted that expressed IL-4. Error bars represent standard deviations (**** = p<0.0001, *** = p<0.001, ** = p<0.01, * = p<0.05).
